# The evolution of penile reconstructive techniques in urology

**DOI:** 10.1038/s41443-025-01141-3

**Published:** 2025-09-10

**Authors:** Mehmet Hamza Gultekin, Abdullah Al-Mitwalli, Wai Gin Lee, David Ralph

**Affiliations:** 1 Department of Urology, Istanbul University - Cerrahpasa, Cerrahpasa Faculty of Medicine, Istanbul, Turkey; 2 Department of Urology, University College London Hospitals, London, United Kingdom

**Keywords:** Erectile dysfunction, Sexual dysfunction, Sexual dysfunction, Tissue engineering, Quality of life

## Abstract

The need to enhance the quality of life and functionality of patients with a number of diseases, such as congenital abnormalities, traumas, and gender incongruence, has contributed to a significant development in the field of male genital reconstructive surgery. This article highlights the roots of penile reconstructive surgeries over history, emphasizing innovative achievements that have shaped modern practices. Critical advancements that have improved surgical accuracy and post-operative care are examined, including new imaging modalities, penile prosthesis implantation, and complete phallic reconstruction. In terms of future improvements in genital reconstructive surgery, the combination of tissue engineering and microsurgery offers the potential to further improve the field.

## Introduction

The evolution of male genital reconstructive surgery has been significantly shaped by the necessity to improve the quality of life and functionality for patients dealing with conditions such as hypospadias, epispadias, urethral strictures, penile curvature, penile cancer, traumatic injuries, congenital abnormalities, and gender incongruence.

Penile reconstructive surgery traces its origins back to ancient procedures such as the surgical treatment of phimosis by the Greek physician Aegineta [1]. The field has seen significant advancements since the efforts of pioneers including Dr. Nikolaj A. Bogoraz [2], Sir Harold Gillies [3], Dr. Reed M. Nesbit [4], and Dr. Ti-Sheng Chang [5] in the late 19^th^ and early 20^th^ centuries. In modern times, these developments have been propelled further by the rising need and numbers of gender affirmation surgery, leading to more refined and effective reconstructive techniques.

As the field progresses, ethical considerations have also drawn more attention, especially in procedures like penile augmentation. Clinical guidelines now emphasize the importance of addressing conditions such as Body Dysmorphic Disorder and Penile Dysmorphic Disorder, which can affect surgical outcomes and patient satisfaction. Therefore, incorporating comprehensive psychological assessments has become vital to ensure safe and successful intervention [6].

This article discusses critical advances across specific areas, including total phallic reconstruction, which focuses on achieving a cosmetically acceptable and functional phallus optimal for both urinary and sexual function. Recent advancements in genital reconstructive surgery also address conditions like Adult-Acquired Buried Penis (AABP), improving functional outcomes and patient satisfaction through refined techniques. It also examines the complexities and pivotal role of penile prosthesis implantation for erectile function, especially following masculinising gender-affirming surgery or in men with Peyronie’s disease with concurrent erectile dysfunction.

Furthermore, we cover the significant advancements in the surgical treatment of penile cancer, noting a shift towards more conservative surgical approaches to better preserve penile function and appearance. The role of advanced imaging modalities, particularly magnetic resonance imaging (MRI), is also discussed, highlighting how they have improved both pre-operative planning and post-operative care in detecting and managing complications, thereby enhancing surgical precision and patient care.

The future of penile reconstructive surgery promises even greater advancements with the integration of cutting-edge technologies such as microsurgical technique and tissue engineering, which aim to further enhance the precision, safety, and outcomes of these complex procedures. The aim of this perspective article is to enrich our understanding of the dynamic field of modern penile reconstruction surgery and its ongoing commitment to technological integration and procedural refinements, giving us clues about what awaits us in the future.

## Advancement in grafting materials

Early reconstructive surgeries were limited by the availability and compatibility of grafting materials. However, synthetic and biocompatible materials have revolutionised the field, offering diverse options to surgeons.

The use of biological materials in reconstructive surgeries has overcome some challenges of synthetic grafts by enhancing biocompatibility and tissue integration. Historically, synthetic polyethylene terephthalate (PETE) and politetrafloroetilen (PTFE) grafts were among the few options but carried risk of infection, inflammatory reaction, and fibrosis [7]. Autologous tissues, like buccal mucosa, saphenous vein, and tunica albuginea, have become preferred grafts for their biocompatibility. ^8^ Additionally, decellular matrix is widely used acting as a scaffold for tissue regeneration [8].

Xenografts and allografts, processed tissues from animal and cadaveric sources, expand options for complex surgeries. Xenografts, from porcine, bovine, etc., mimic human tissue structure, enhance tissue integration and promote cellular ingrowth [9]. However, resorption rates vary, potentially affecting the long-term success and stability of surgical repairs [10]. Faster resorption might compromise structural support, while delayed resorption might lead to fibrosis, affecting natural feel and flexibility [10].

Significant advancements in tissue processing such as chemical cross-linking and decellularization techniques, date back to the 1960s. These developments improved biocompatibility and reduced antigenicity, thereby enhancing graft success rates [11]. These developments set the stage for introducing porcine small intestinal submucosa and pericardial grafts, which supports cell proliferation, tissue remodelling, and vascularization at the graft site, in reconstructive urology [12, 13]. TachoSil ^®^ (Corza Medical, MA, USA), an equine collagen fleece with human fibrinogen and thrombin, promotes haemostasis and wound healing, particularly useful in Peyronie’s disease correction [14]. Building on these developments, acellular dermal matrices (ADMs) have become as transformative tools for reconstructing donor sites, providing superior healing, aesthetics, and lower morbidity compared to traditional grafts (split and full thickness skin grafts) [15]. ADMs, used in combination with split-thickness skin grafts, demonstrate higher graft take rates (93.8% vs. 27.8%, *p* = 0.001), faster healing times (24 days vs. 30 days, *p* = 0.003), shorter operative durations, and greater patient satisfaction compared to full-thickness skin grafts, especially in procedures like radial forearm free flap phalloplasty [15–17]. These developments exemplify the diversification and refinement of xenograft materials, including hybrid materials integrating bioactive molecules or stem cells.

## Advancements in penile straightening procedures

The evolution to contemporary penile straightening procedures reflects an improved understanding Peyronie’s disease, innovative techniques, improved grafting materials.

Early surgical attempts to correct penile curvature began in the late 19th century with plaque excision by MacClellan, Regnoli and Huitfeldt [18]. These were isolated cases and lacked standardization [18]. By the early 20^th^ century, the treatment of this disease involved plaque excision followed by the placement of dermal grafts, fat, or fascia over defect to restore penile function and appearance. Lowsley and Boyce reported 60% success rate but also over 20% of recurrence rate among patients with this technique [19].

**Fig. 1:**
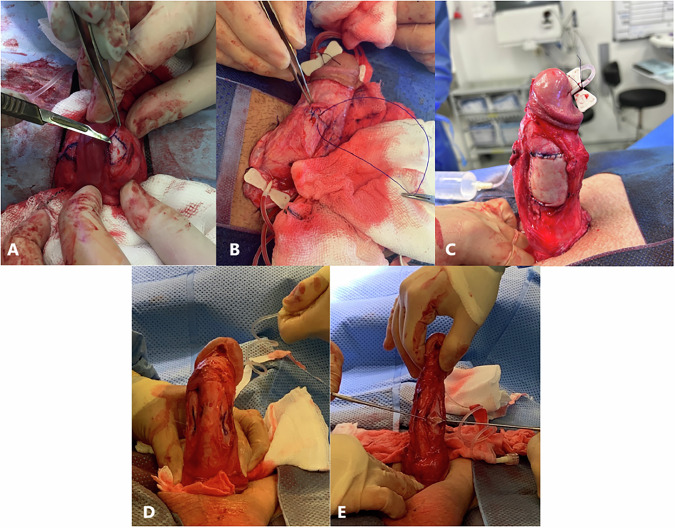
Examples of surgical techniques for penile curvature correction. **A** Attenuated Nesbit procedure in a patient with dorsal curvature, **B** Plication procedure in a patient with left-sided curvature, **C** Lue Procedure of a patient with dorsal side curvature. Artificial erection confirms the correction of the penis, **D** Curvature assessment with artificial erection, **E** Left-sided vertical incision of ventral surface of the left corporeal body of the penis. This incision will be closed in a transvers fashion afterward.

A breakthrough advance occurred in 1965 with the Nesbit’s procedure [4]. This technique became foundational for curvature correction. The procedure involves excision and plication of the tunica albuginea opposite the curvature, straightening the penis (Fig. 1A).

Following the Nesbit’s procedure, various modifications emerged to reduce complications. Less invasive techniques like the Essed-Schroder plication [20] and the Lue 16-dot technique [21], introduced plications without tunica albuginea excision, reducing the risk of erectile dysfunction (Fig. 1B) Additionally, rotation techniques that cause minimal shortening of the penis length in the ventral curvatures have also been described [22].

For severe cases of Peyronie’s disease with significant curvature or risk of significant shortening with plication, another technique is to incise or excise the plaque and use grafts (autolog, allograft, xenograft, or synthetic) to fill the defects (Fig. 1C).

The Yachia technique modified the Nesbit procedure by using longitudinal incisions with transverse closures (Heineke-Mikulicz method), reducing erectile dysfunction risk and shortening operative time [23] (Fig. 1D, E).

Additionally, traction and vacuum devices gained popularity to reducing curvature and restoring penile length. Their increasing use indicates a growing preference for non-surgical methods [24].

In recent decades, minimally invasive treatment such as collagenase Clostridium Histolyticum injections have shown promise by dissolving collagen accumulations causing curvature and reducing the need for surgery [25]. Furthermore, Extracorporeal Shock Wave Treatment has also been explored as a treatment for Peyronie’s disease pain relief, while its efficacy in curvature remains uncertain [26].

Looking forward, research in tissue engineering and stem cell therapy, could improve both invasive and non-invasive therapies for penile deviation and Peyronie’s disease. These advancements appear promising for new methods to repair or regenerate tunica albuginea tissue, potentially reducing the need for surgery and enhance outcomes.

## Phalloplasty techniques: evolution and current trends

The concept of phalloplasty dates back to the early 20^th^ century, with initial procedures focused on treating injuries and congenital defects [2]. Early surgeries were rudimentary and primarily aimed to create a structure that resembled a penis, with limited focus on functionality or aesthetics [2].

**Fig. 2:**
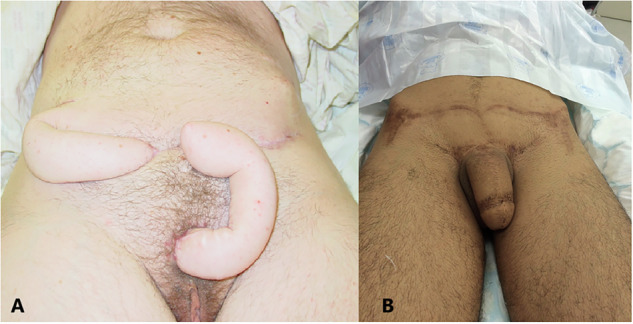
Techniques of phalloplasty in gender-affirming surgery. **A** Gillies phalloplasty and **B** abdominal phalloplasty.

In the mid-20th century, surgeons used tubed pedicle grafts, harvested from areas like the thigh, abdomen, or forearm, to construct a penis [2, 3]. In order to create the phallic structure, a skin graft was rolled into a tube and affixed to the pubic area. While this represented a significant advancement, results often lacked in appearance, sensation, and function [3, 27] (Fig. 2A, B).

**Fig. 3:**
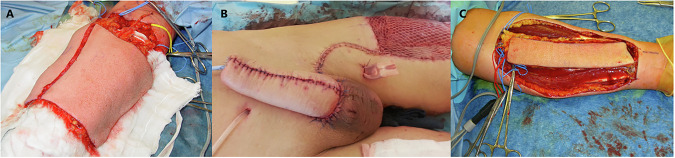
Examples of flap techniques used in phalloplasty. **A** Radial forearm free flap, **B** anterolateral thigh (ALT) flap, and **C** radial forearm free flap urethra.

The introduction of microsurgery in the 1980s revolutionized phalloplasty with free flap techniques. The radial artery forearm free-flap (RAFFF) has gained popularity, allowing for the transferring skin, fat, nerve, and vascular tissue from the forearm to create functional and natural-looking results [5]. This technique improved standing micturition and sensation prospects [5] (Fig. 3A).

Free flap techniques evolved with alternative donor sites, like the Anterolateral Thigh (ALT) and the Latissimus Dorsi (LD), reducing morbidity and enhancing aesthetics [28, 29] (Fig. 3B).

There are also improvements in the creation of the neo-urethra, allowing for more reliable standing micturition [30]. RAFFF urethral reconstruction gains popularity due to its benefit of introducing reliable, well-vascularized, and sensitive tissue [31] (Fig. 3C).

The ideal phalloplasty goal is to create a cosmetically acceptable and sensate phallus with an integrated neourethra, allowing the patient to void while standing and adequate bulk to accommodate a stiffener for sufficient rigidity for intercourse [32]. Stiffness techniques have included osteocutaneous flaps cartilage insertion during initial surgeries, but they often led to complications including rigidity issues, infection, and challenges in achieving and maintaining a natural appearance and functionality [33].

Implanting penile prostheses, especially inflatable types, has become an essential aspect of phalloplasty. Since Puckett and Montie introduced penile implantation in phalloplasty for gender dysphoria in 1978, the procedure has significantly improved functionality for transgender men [34]. However, it is acknowledged as complex with high complication rates, necessitating experienced surgeons in dedicated, high-volume centres [35]. Furthermore, testicular implants and scrotoplasty techniques have also seen advancements, enabling more precise reconstructions [36–38]. Recent advancements in scrotoplasty have introduced techniques such as the V-Y advancement of the labia majora and the Hoebeke method, which improve the anatomical and aesthetic outcomes of neoscrotal construction [36, 37]. Additionally, testicular prostheses, often implanted in a following procedure, now better replicate the size and shape of biological testicles, offering enhanced cosmetic and psychological benefits, though long-term clinical outcomes remain under-documented [38].

Advanced technologies, such as robotics and 3D printing, could improve phalloplasty techniques, particularly for phallus shaping [39, 40]. Emerging tissue engineering and regenerative medicine could eventually generate enable lab-grown penile tissue for transplantation [8]. Though still in their early phases, these methods hold promise for individuals needing sophisticated male genital reconstructive surgeries.

## Advancements in penile cancer surgeries

The incidence of penile carcinoma has risen recently, although it is still rare, accounting for <1% of all male cancers. In the United Kingdom, there are around 350 new diagnoses of penile cancer annually, with an incidence of 1.1 per 100,000 [41].

Surgical treatments of penile cancer have evolved significantly, breakthroughs led by research looking at positive surgical margins and local recurrence rates, which has caused advancements over the years, focusing primarily on improving patient outcomes and preserving penile function [42].

**Fig. 4:**
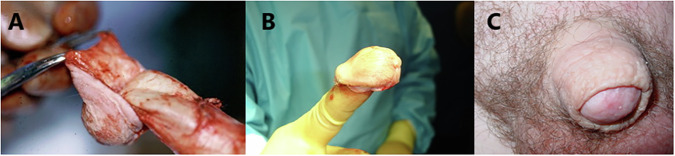
Conservative surgical approaches in penile cancer treatment emphasizing tissue preservation. **A** Perioperative view of glansectomy. **B** Completely removed glans penis, **C** Postoperative view of pseudo glans formation.

Historically, extensive resections, including partial or total penectomy were common, severely impacting quality of life, particularly sexual function [43]. While amputation remains necessary in some cases, more conservative methods are now favoured, with safety margins progressively shortened [44]. Earlier practices required around 2 cm margins, but recent studies have demonstrated that even a few millimetres free margin can provide effective outcomes, preserving more healthy tissue and improving penile function and appearance [44] (Fig. 4A–C).

Penile preserving techniques, now a well-documented treatment for Tis, T1, and T2 tumours, offering better sexual and urinary function without compromising oncological safety or cancer-specific survival [45]. Advancements in reconstructive techniques, like split skin grafts, further enhance functional and cosmetic outcomes [42].

Looking forward, advancements in robotic and laser surgery, promise greater accuracy and less invasiveness [46, 47]. Emerging topical and intralesional treatments that will lessen the need for major surgeries [48]. These developments indicate a shift toward organ preservation and improved patient quality of life.

## Adult-acquired buried penis (AABP): evolution of surgical techniques

The surgical management of AABP, a condition increasingly linked with the global rise in obesity [49], has evolved significantly in recent years [50]. AABP was an unappreciated condition in the past, leading to severe functional impairments in urinary and sexual function, psychological distress, and a worse quality of life [51]. Modern surgical developments have aimed to address these complex problems.

Recent studies highlight various techniques for AABP repair, including escutcheonectomy with or without skin grafting, liposuction, V-Y plasty, scrotoplasty, penoplasty with diamond-shaped incisions and concurrent panniculectomy with fascial reconstruction [50, 52–54]. According to Wang et al., “diamond-shaped penoplasty” combined with suprapubic liposuction have demonstrated enhanced penile length and aesthetic outcomes with little side effects [52]. The use of panniculectomy in conjunction with AABP repair has also improved functional and psychological results with similar rates of complications [53]. Additionally, split-thickness and full-thickness skin grafts used for AABP repair did not significantly differ in surgical, functional, or patient-reported outcomes, based on a recent comparative retrospective study by Gül et al., highlighting the benefits of both approaches in managing cases involving significant penile skin loss [55]. In order to enhance postoperative support and aesthetics, anatomic studies have refined fascial reconstruction including reapproximating the fundiform ligament [54].

Despite difficulties, particularly with obese patients, AABP surgery has changed as a result of developments in reconstruction, soft tissue excision, and grafting. These methods show the value of cooperation between urologic and plastic surgeons by restoring function and reducing the psychological and social problems associated with AABP. Long-term outcomes and patient satisfaction are the goals of continuous enhancements.

## The role of imaging modalities in penile reconstruction

Imaging modalities are essential in male genital reconstructive surgery, serving diagnosis, pre-operative planning, and post-operative care. Techniques such as ultrasound, computed tomography (CT), and particularly MRI offer vital insights into anatomical structures and clinical situations (Supplementary Figure).

Recognized for its detailed anatomical visualizations, MRI’s significance in medical diagnostics was initially established in the late 1980s [56]. It has proven highly effective at revealing normal anatomical features and various abnormalities such as congenital anomalies, penile prostheses complications, trauma-related alterations, Peyronie’s disease, and neoplasms, thereby significantly aiding in diagnosis and surgical planning [57]. MRI demonstrates outstanding performance across a wide range of genitourinary complex scenarios, staging penile cancer when physical examination results are unclear, assessing tissue viability in priapism, determining injuries in penile fractures, aiding in surgical planning for penile fibrosis and Peyronie’s disease, and potentially replacing urethrography in complex pelvic fractures [57, 58]. Furthermore, MRI plays a critical role in the management of complications in penile prosthesis, evaluating the positioning, configuration, and functional state of malleable and inflatable prostheses, and detecting problems like infection, mechanical failure, and malpositioning [59].

Future advances may include advanced MRI techniques, augmented reality for real-time surgical navigation, and machine learning algorithms to enhance surgical accuracy and patient outcomes.

## Conclusion

Significant advancements in male genital reconstructive surgery have improved patient outcomes and expanded treatment options through materials ranging from synthetic, biocompatible substances to tissue-engineered solutions and natural biological materials like xenografts. These materials, consist of porcine, bovine, and equine tissues, have structural benefits and affordability despite immunological responses and disease transmission risk. Innovations like TachoSil^®^ demonstrate dedication to raising the standard and effectiveness of reconstructive surgeries. Meanwhile, the field of penile straightening has evolved toward refined, less invasive techniques, that preserve sexual function, reflecting a deeper understanding of conditions like Peyronie’s disease. Additionally, advancements in phalloplasty have paralleled these improvements, evolving from tubed pedicle grafts to sophisticated free flap microsurgical techniques that enhance structural, functional, and cosmetic results. While the evolution of penile prosthesis surgeries, particularly for patients undergoing phalloplasty, has achieved greater functionality, and patient satisfaction. Furthermore, penile cancer surgeries emphasize conservative approaches prioritizing organ function and preservation. The future of these reconstructive treatments looks promising with advancements in regenerative medicine, offering potential to overcome current limitations.

## Supplementary information

Supplementary Figure Legends

Supplementary Figure 1.png

Supplementary Figure 2.png
