# Supplementary material for: The evolution of penile reconstructive techniques in urology
Source: Int J Impot Res. 2025 Sep 10;38(3):155–60. doi: 10.1038/s41443-025-01141-3 (PMC12999490; doi:10.1038/s41443-025-01141-3)
Supplement: Supplementary file 1 — Supplementary Figure Legends [file 41443_2025_1141_MOESM1_ESM.docx]

**Supplementary Figure 1: Cylinder extrusion appearance on MRI. Bilateral cylinders are seen outside the corporeal bodies on coronal T2-weighted image.**

**Supplementary Figure 2: Penile Reconstructive Surgery Timeline; Key surgeries and researches.**
