# Supplementary figures and images for: The evolution of penile reconstructive techniques in urology
Source: Int J Impot Res. 2025 Sep 10;38(3):155–60. doi: 10.1038/s41443-025-01141-3 (PMC12999490; doi:10.1038/s41443-025-01141-3)

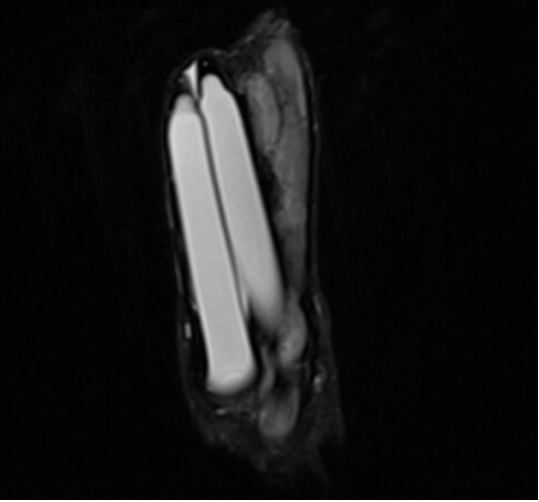

Supplement: Supplementary file 2 — Supplementary Figure 1.png [file 41443_2025_1141_MOESM2_ESM.png]

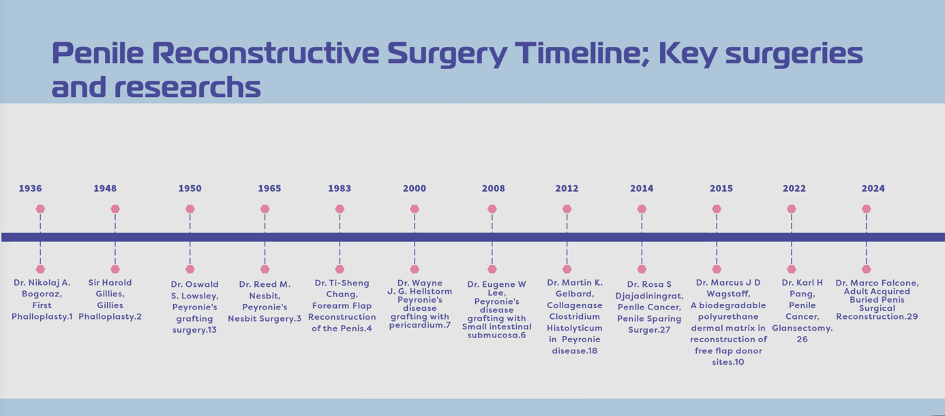

Supplement: Supplementary file 3 — Supplementary Figure 2.png [file 41443_2025_1141_MOESM3_ESM.png]
